# Post‐traumatic cerebral fat embolism syndrome with a favourable outcome: a case report

**DOI:** 10.1186/s12883-021-02076-0

**Published:** 2021-02-18

**Authors:** Wei Wang, Weibi Chen, Yan Zhang, Yingying Su, Yuping Wang

**Affiliations:** Department of Neurology, Xuanwu Hospital, Capital Medical University, 45th Changchun Street, 100053 Beijing, China

**Keywords:** Fat embolism syndrome, Neurological manifestations, Cerebral fat embolism, Case report

## Abstract

**Background:**

Fat embolism syndrome (FES) is a change in physiology resulting from mechanical causes, trauma, or sepsis. Neurological manifestations of FES can vary from mild cognitive changes to coma and even cerebral oedema and brain death. Here, we present an unusual case of cerebral fat emboli that occurred in the absence of acute chest syndrome or right-to-left shunt.

**Case presentation:**

A previously healthy 57-year-old right-handed male was admitted to our department because of unconsciousness after a car accident for 3 days. He suffered from multiple fractures of the bilateral lower extremities and pelvis. This patient had severe anaemia and thrombocytopenia. Head MRI showed multiple small lesions in the whole brain consistent with a “star field” pattern, including high signals on T2-weighted (T2w) and fluid-attenuated inversion recovery (FLAIR) images in the bilateral centrum semiovale; both frontal, parietal and occipital lobes; and brainstem, cerebellar hemisphere, and deep and subcortical white matter. Intravenous methylprednisolone, heparin, mannitol, antibiotics and nutritional support were used. Although this patient had severe symptoms at first, the outcome was favourable.

**Conclusions:**

When patients have long bone and pelvic fractures, multiple bone fractures and deteriorated neurological status, cerebral fat embolism (CFE) should be considered. Additionally, CFE may occur without an intracardiac shunt. The early diagnosis and appropriate management of FES are important, and prior to and following surgery, patients should be monitored comprehensively in the intensive care unit. With appropriate treatment, CFE patients may achieve good results.

## Background

Fat embolism syndrome (FES) is a change in physiology resulting from mechanical causes, trauma, or sepsis. Fat globules generated within the systemic circulation induce pulmonary dysfunction, neurological changes, dermal symptoms, and dysfunction of several other organs. Although very rare, FES is a fatal disease that develops within 12–72 hours [1, 2]. The neurological manifestations of FES can vary from mild cognitive changes to coma and even cerebral oedema and brain death. On magnetic resonance imaging (MRI), cerebral fat embolism (CFE) can demonstrate a “star field” pattern due to the presence of multiple microembolic infarcts in the whole brain [3]. Here, we report a case of isolated CFE, and no pulmonary dysfunction or intracardiac shunt was found. Although this patient had severe symptoms at first, the outcome was favourable.

## Case presentation

A previously healthy 57-year-old right-handed male was admitted to our department because of unconsciousness after a car accident for 3 days. Three days before admission, this patient had a car accident and suffered from multiple fractures of the bilateral lower extremities and pelvis. Then, he was sent to the local emergency centre and underwent external fixation surgery for the fractures. Before surgery, this patient was conscious at all times, even though he experienced hypotensive shock (blood pressure decreased to 80/50 mmHg, heart rate: 110–120 times/min) and received blood transfusion and other rescue measures. This patient was transported to the recovery room after surgery. During the anaesthesia recovery period, the sedation and analgesia drugs were continued after the patient became conscious. Then, this patient lost consciousness and could no longer be roused. Head MRI showed multiple small lesions in the whole brain, and fat embolism was taken into consideration. A chest computed tomography (CT) scan showed bilateral lung contusion. 3D pulmonary arteriography with contrast was also performed to rule out pulmonary embolism and lung vascular shunt. The patient was considered to be suffering from CFE, and intravenous methylprednisolone and albumin were used for three days, but no improvement was shown.

Then, this patient was transferred to the intensive care unit (ICU) of our hospital. His vital signs were stable (T: 37.3℃, P: 108/min, R: 21/min, BP: 122/84 mmHg). On neurological examination, he was in coma. His bilateral pupillary reflex was normal, and his oculocephalic, corneal and cough reflexes existed. Pain stimuli in this patient showed slight flexion of the bilateral limbs. The bilateral Babinski sign of this patient was positive. Written informed consent was obtained from the legal guardians of this patient.

This patient had severe anaemia. The routine blood test showed that the lowest red blood cell (RBC) count was 1.70*10^12^/L, and the lowest haemoglobin (HGB) level was 72 g/L. He also had fever (highest temperature was 38.5°C) and thrombocytopenia (the lowest platelet count was 67*10^9^/L) during the course of the disease. The D-dimer and fibrinogen levels of this patient were higher than normal levels for more than one month before they reached normal ranges (the highest D-dimer level was 47.3 µg/ml, and the highest fibrinogen level was 115.4 g/L).

**Fig. 1:**
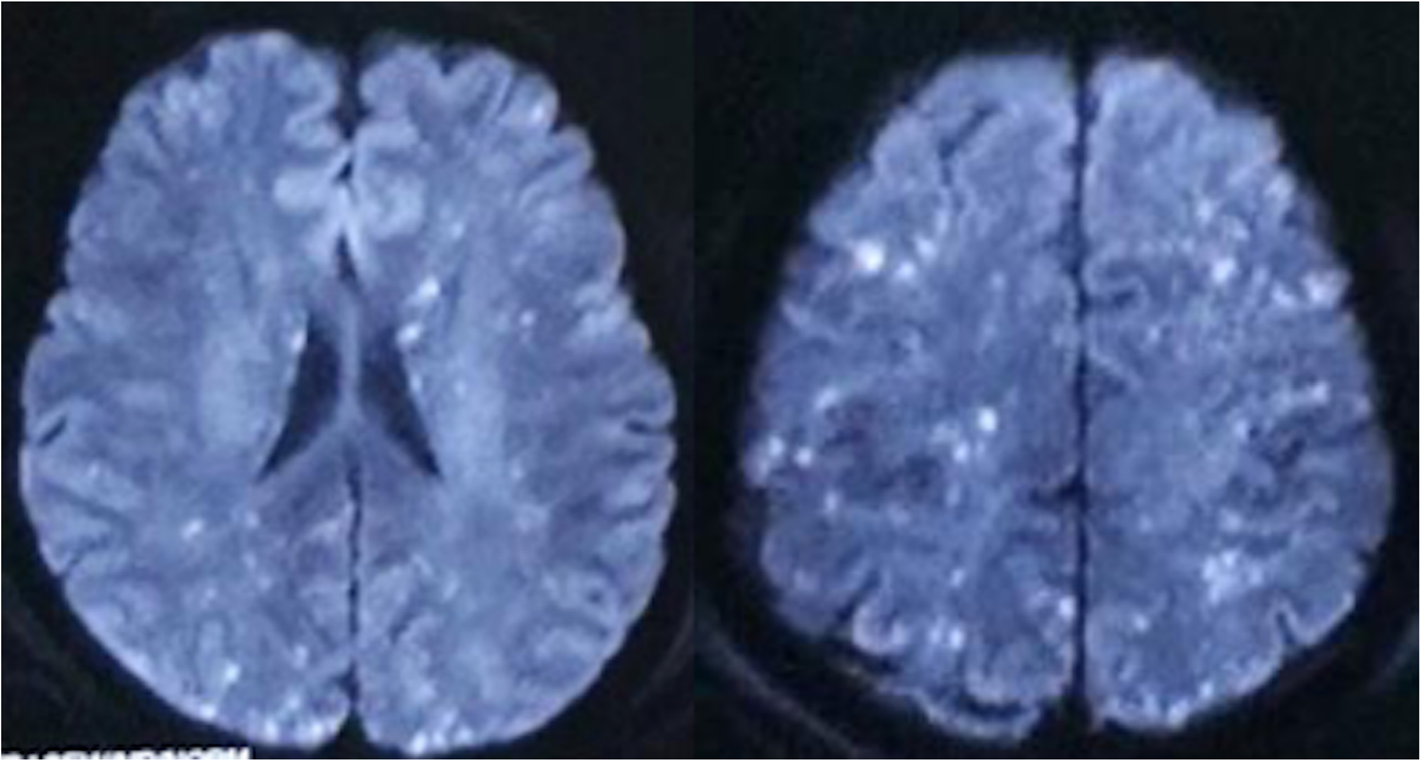
Head MRI in the local emergency centre showed whole brain multiple small lesions consistent with “star field” pattern

**Fig. 2:**
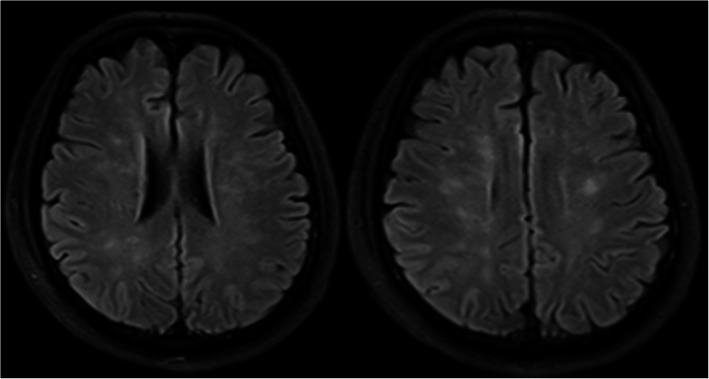
Head MRI repeated 15 days after the previous head MRI showed the signals of the lesions on T2w and FLAIR images were slightly decreased

Bilateral lower extremity X-ray and pelvic CT in this patient showed multiple fractures of the bilateral lower extremities and pelvis. Head MRI showed multiple small lesions in the whole brain consistent with a “star field” pattern (Fig. 1), including high signals on T2-weighted (T2w) and fluid-attenuated inversion recovery (FLAIR) images in the bilateral centrum semiovale; both frontal, parietal and occipital lobes; and brainstem, cerebellar hemisphere, and deep and subcortical white matter. Head MRI was re-examined 15 days after admission to our hospital and revealed that the signals of these lesions on T2w and FLAIR images were slightly decreased (Fig. 2). Continuous electroencephalogram (EEG) monitoring showed generalized slow waves without epileptiform discharges. Bedside transthoracic echocardiography of this patient was highly suspicious of a patent foramen ovale. However, the transesophageal echocardiogram with bubble study failed to demonstrate an intracardiac defect or arteriovenous malformation (AVM) in the lung, further supporting a biochemical process.

In terms of the treatment, intravenous methylprednisolone was used for 9 days in total (500 mg *1 day, 1 g*2 days, 500 mg*3 days, 80 mg*3 days), including the first 3 days prescript in the local emergency centre; heparin was used for 8 days until side effects occurred (right leg haematoma); and mannitol, antibiotics and nutritional support were also used simultaneously.

**Fig. 3:**
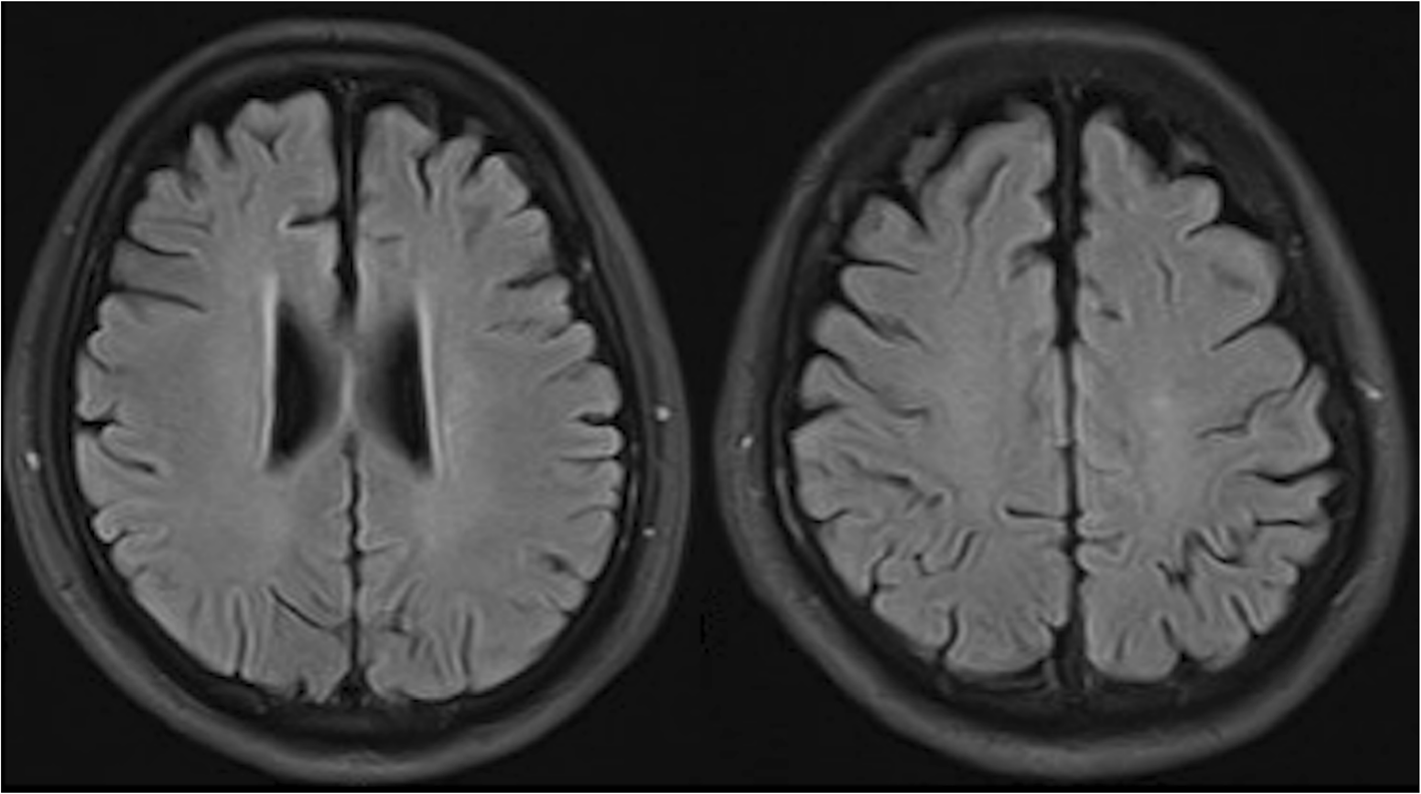
Head MRI of this patient re-examined 11 months after the car accident showed that the “star field” pattern lesions were almost completely absorbed

Four days after admission, the mechanical ventilator was removed, and the blood oxygen saturation of this patient was normal. After this treatment combination was administered for 33 days, the patient’s sensorium improved with eye opening in response to painful stimuli. Forty-six days later, he could open his eyes and had some tracking eye movement. He could open his eyes to verbal stimuli and perform some simple body movements, but he did not speak coherently. Fifty-five days later, his consciousness gradually became normal, and he underwent internal fixation surgery for a right lower limb fracture. Figure 3 shows his head MRI results 11 months after the car accident, and the lesions were almost completely absorbed. The Barthel index was 95 at the last visit 10 months later.

## Discussion and conclusion

FES is characterized by both major and minor findings following long bone trauma and/or major orthopaedic procedures as defined by Gurd [4]. The major criteria include hypoxia, deteriorating mental status, and petechiae. The minor criteria consist of tachycardia, fever, anaemia, and thrombocytopenia. To meet the clinical diagnosis of FES, patients need to demonstrate at least 1 major and 3 minor or 2 major and 2 minor signs.

FES is caused by fractures of the ilium or other pelvic bones and multiple traumas [5]. However, other possible causes include severe burns, infection, kidney transplant, liposuction, cardiopulmonary bypass, and transfusions. Some rare causes, such as gastrectomy or hepatocellular carcinoma, have also been reported [6–9]. The incidence of post-traumatic FES has been reported to be as low as 0.9–2.2 % in retrospective studies and as high as 35 % in prospective studies [10–12].

The clinical onset of symptoms may occur within 12 hours, but symptoms usually present 24–72 hours later. The presentation is variable, and no individual symptom is diagnostic of the syndrome. Patients with FES present with a classic triad of respiratory manifestations (95 %), cerebral effects (60 %) and petechiae (33 %) [13, 14]. Neurological signs due to cerebral emboli occur in up to 86 % of cases and often occur after the development of respiratory distress. Neurological manifestations of FES can vary from mild cognitive changes to coma and even cerebral oedema and brain death [15]. Petechial rash is considered pathognomonic of FES and is reportedly present in up to 60 % of patients, usually on the conjunctiva, oral mucous membranes, skin folds of the neck and axillae [16]. Although neurological symptoms are typically accompanied by respiratory failure and skin eruptions, isolated cerebral fat embolism cases have been reported in several instances [17, 18]. In our case, detailed respiratory system and skin examinations were normal. The main initial symptom was the disturbance of consciousness.

One study reviewed 1692 patients with long bone and pelvic fractures and found that 12 patients (0.7 %) met the diagnosis of FES. Five had multiple bone fractures (42 %), and three were diagnosed with CFE. All of the patients with CFE had neurological status alterations and showed T2 and FLAIR hyperintense lesions in the bilateral cerebral hemisphere, basal ganglia, thalamus, pons and cerebellum [10]. Despite the coma for almost 2 months, repeated MRI in our patient showed resolution of the lesions. Although the initial presentation of CFE may be severe, the majority of case reports on CFE illustrate that the cerebral dysfunction associated with CFE is reversible [12]..

Radiological findings are useful for the diagnosis of CFE. Brain CT shows normal findings in most cases. Brain MRI is more sensitive and can show small high signal intensity lesions scattered in the cerebral white matter, cerebellum, and brainstem on T2-weighted images or diffusion-weighted images [7, 19].

The pathogenesis of FES is unclear but is thought to involve mechanical obstruction and biochemical injury [15, 20, 21]. The mechanical theory postulates that fat microemboli enter venous sinusoids, collect in the pulmonary microvasculature, and occasionally migrate into the systemic circulation via the pulmonary capillary bed or right-to-left shunt. The incidence of intracardiac shunt has been described to occur in 20–34 % of the population. Additionally, micro-fat droplets can theoretically traverse the pulmonary circulation without sequestration, resulting in systemic symptoms. Neuronal ischaemia followed by cytotoxic oedema occurs in most patients with cerebral fat embolism. Ischaemic changes typically occur in watershed areas, seen as a “star field” pattern [11]. Cerebral fat emboli can often cause the brain to appear oedematous and demonstrate an inflammatory reaction, while numerous petechiae can cover the surface of the brain. It has been hypothesized that the volume of diffusion restriction on initial MRI may be able to predict outcomes [22, 23].Fulminant fat embolism is characterized by occlusion of the microvasculature via fat emboli, resulting in microinfarction and haemorrhage [1, 18, 24].

The biochemical theory postulates that plasma mediators cause a systemic release of free fatty acids, causing inflammation and endothelial damage [21, 25]. Previous reports have hypothesized that inflammatory reactants, including lipoprotein lipase, cause the release of fatty acids, thus altering the fat transport mechanisms of plasma. This change in homeostasis results in fat droplet aggregation with systemic sequestration in the microvasculature. These emboli exacerbate the development of organ dysfunction. Our patient did not develop an acute chest syndrome, and chest CT was negative for fat emboli, eliminating an obvious pulmonary origin. Additionally, the work-up was negative for shunting, suggesting that the development of cerebral fat emboli may have been mediated by a biochemical rather than a mechanical process.

In FES, prevention, early detection, and appropriate treatment are important. First, anaesthesiologists and surgeons should recognize patients at risk for developing fat embolism syndrome. Some case reports have suggested that the use of intracranial pressure monitoring and cerebral tissue oxygenation monitoring clearly defines neuro-protective targets for optimum perfusion, and intracranial pressure control is helpful [24]. Supportive care continues to be the mainstay of treatment for FES. Multiple treatment options have been evaluated in the past without significantly changing clinical outcomes, including clofibrate, dextran-40, ethyl alcohol, heparin, aspirin, human albumin and steroids [26–28]. Early splinting and fixation of orthopaedic fractures improve outcomes in trauma patients [27, 29].

Most patients of FES recover completely if adequate supportive treatment and improved nursing is provided. The overall mortality for this condition is 5–15 %, with severity of respiratory problems being a close indicator of the risk of death [2]. The majority of case reports on CFE illustrate that cerebral dysfunction associated with CFE is reversible [3, 12, 30, 31].

In conclusion, clinically apparent FES is unusual and requires a high index of suspicion, especially in long bone and pelvic fractures. When patients have long bone and pelvic fractures, multiple bone fractures or deteriorated neurological status, CFE should be considered. Additionally, CFE may occur without an intracardiac shunt. To prevent FES, risk factors should be corrected if possible, and careful anaesthetic management should be undertaken. Furthermore, the early diagnosis and appropriate management of FES are important, and prior to and following surgery, patients should be monitored comprehensively in the intensive care unit. With appropriate treatment, CFE patients may achieve good results.

## Data Availability

All data generated or analysed during this study are included in this published article.

## Abbreviations

AVM: Arteriovenous malformation
CFE: Cerebral fat embolism
CT: Computed tomography
EEG: Electroencephalogram
FES: Fat embolism syndrome
FLAIR: Fluid attenuated inversion recovery
HGB: Hemoglobin
ICU: Intensive care unit
MRI: Magnetic resonance imaging
RBC: Red blood cell
T2w: T2-weighted
