# Correction to: Post-traumatic cerebral fat embolism syndrome with a favourable outcome: a case report

**DOI:** 10.1186/s12883-021-02211-x

**Published:** 2021-05-11

**Authors:** Wei Wang, Weibi Chen, Yan Zhang, Yingying Su, Yuping Wang

**Affiliations:** Department of Neurology, Xuanwu Hospital, Capital Medical University, 45th Changchun Street, Beijing, 100053 China

**Correction to: BMC Neurol 21, 82 (2021)**

**https://doi.org/10.1186/s12883-021-02076-0**

Following publication of the original article [1], the authors reported an error in the contribution of the authors, Dr. Wei Wang and Dr. Weibi Chen contributed equally to this work.

The original article has been corrected.
